# Establishment and validation of a prognostic model for nasopharyngeal carcinoma patients based on partial response rates

**DOI:** 10.3389/fonc.2025.1705634

**Published:** 2025-11-19

**Authors:** Fujue Wang, Qiao Yang, Dong Yang, Chuangjie Cao, Xinghua Chen, Jiancheng Ning, Tianyu Wu, Wei Zhou, Zhe Fang, Pian Li

**Affiliations:** 1 State Key Laboratory of Biotherapy and Cancer Center, West China Hospital, Sichuan University, Chengdu, Sichuan, China; 2 Department of Hematology, The First Affiliated Hospital of Hengyang Medical School, University of South China, Hengyang, Hunan, China; 3 Department of Oncology, The First Affiliated Hospital of Hengyang Medical School, University of South China, Hengyang, Hunan, China; 4 Department of Pathology, The First Affiliated Hospital of Hengyang Medical School, University of South China, Hengyang, Hunan, China; 5 Department of Radiation Oncology, The First Affiliated Hospital of Guangxi Medical University, Nanning, Guangxi, China; 6 School of Chinese Medicine, Hunan University of Chinese Medicine, Changsha, Hunan, China

**Keywords:** nasopharyngeal carcinoma (NPC), partial response (PR), induction chemotherapy (IC), nomogram, prognosis

## Abstract

**Objective:**

This study aims to investigate the impact of varying rates of partial response (PR) on survival outcomes in nasopharyngeal carcinoma (NPC) patients following induction chemotherapy (IC) and to develop a nomogram for predicting overall survival (OS).

**Methods:**

Clinical data from 561 NPC patients with PR after IC at two institutions between 2014 and 2019 were analyzed using Cox regression. A nomogram was developed and assessed with the concordance index (C-index), calibration curves, Receiver Operating Characteristic (ROC) curves, and Decision Curve Analysis (DCA). Patients were stratified into risk groups based on nomogram scores, followed by the subgroup analyses.

**Results:**

Age, M stage, primary tumor volume post-IC, cervical lymph nodes volume post-IC, lymphocyte-to-monocyte ratio (LMR), and PR rate were independent OS predictor for NPC patients. The nomogram showed strong discrimination (C-index: 0.769) and outperformed TNM staging in predicting OS. The nomogram’s risk scores effectively stratified patients into high- and low-risk groups, with low-risk patients had better OS, progression‐free survival (PFS) and distant metastasis-free survival (DMFS). Subgroup analyses revealed a significant association between the cumulative dose of cisplatin chemotherapy and survival outcomes in patients with a PR rate below 49%. For those with a PR rate above 49%, cervical lymph nodes volume and the LMR were independent prognostic factors after IC.

**Conclusion:**

We developed and validated a nomogram that predicts the OS of NPC patients undergoing induction chemotherapy based on their PR rates. This tool helps clinicians forecast patient survival. Additionally, it provides valuable insights for optimizing treatment strategies.

## Introduction

Nasopharyngeal carcinoma (NPC) is distinct from other head and neck squamous cell carcinomas in its unique epidemiological pattern, characterized by a markedly higher incidence in southern China and Southeast Asia, and its divergent clinical behavior and therapeutic responsiveness (1, 2). Currently, the main known causes of NPC include Epstein-Barr virus (EBV) infection, chemical carcinogens, environmental factors, and genetic predispositions (3). With advancements in diagnostic and therapeutic techniques, the survival rate for NPC has gradually improved. The standard treatment for locally advanced NPC remains radiotherapy combined with chemotherapy; however, immunotherapy is increasingly being integrated into comprehensive treatment strategies as part of ongoing clinical development (4). Because the nasopharynx is located in a concealed anatomical area, about 80% to 90% of NPC cases are diagnosed at intermediate to advanced stages of the disease. The predominant histological types are poorly differentiated or undifferentiated carcinomas, both characterized by high malignancy and a significant incidence of recurrence and metastasis (5). These factors contribute significantly to treatment failure. Adding induction chemotherapy to the treatment regimen for locally advanced NPC has shown benefits for patients (6). However, recurrence and metastasis lead to mortality in 20% to 30% of patients (7). Thus, there is an urgent need to develop more effective treatment strategies that are tailored to patients with varying risk profiles.

The TNM classification system is widely used for cancer staging and treatment decisions, particularly for assessing risk and stratifying treatment in NPC patients (8). However, the TNM staging system may not accurately predict the prognosis of NPC. Recent clinical studies have shown that clinical factors related to NPC, such as Epstein-Barr virus (EBV) DNA levels, age, and gender, significantly correlate with patient prognosis (9–11). Furthermore, reports indicate that preoperative blood tests, including inflammatory markers, can provide valuable prognostic insights (12). Additionally, the type of treatment, including the number of cycles and specific induction chemotherapy regimens, is prognostically relevant (13, 14), and tumor response to chemotherapy was closely linked to the prognosis of NPC patients (15).

According to the Response Evaluation Criteria in Solid Tumors (RECIST) version 1.1, tumor responses are classified into four categories: complete response (CR), partial response (PR), stable disease (SD), and progressive disease (PD) (16). Patients who achieve CR or PR are more likely to have a better prognosis compared to those with PD or SD after IC (17). RECIST defines a partial response (PR) as a reduction in tumor size of at least 30%. Many patients show varying levels of PR following induction therapy. To date, it has not been examined whether variations in remission rates influence the prognosis of patients with NPC.

Nomograms are useful tools that combine various risk factors into a simple graphical model to predict patient outcomes. This study aims to examine how different PR rates after induction chemotherapy affect the survival outcomes of patients with NPC. Additionally, we conducted a comprehensive analysis and created a nomogram to guide personalized treatment strategies for patients with NPC, considering different risk strata.

## Methods

### Patient screening

We gathered data on NPC patients from January 2014 to December 2019 at two institutions. Patients from The First Affiliated Hospital of Guangxi Medical University were randomly split into a training cohort and an internal validation cohort using the “caret” R package, while those from The First Affiliated Hospital of Hengyang Medical School, University of South China, were employed in the external validation cohort. The inclusion criteria included: i) Patients showing a partial response (PR) to induction chemotherapy, as defined by a reduction of at least 30% in the longest diameter of measurable lesions based on RECIST 1.1; ii) Disease classified as stages II, III, IVA, or IVB according to the 8th edition of the American Joint Committee on Cancer (AJCC) staging system; iii) Previous induction chemotherapy for all patients; iv) Completion of computed tomography (CT) or magnetic resonance imaging (MRI) of the head and neck before and after induction chemotherapy; v) Presence of at least one measurable lesion meeting the RECIST v1.1 criteria. The exclusion criteria were patients who: i) had a history of other malignancies; ii) had severe comorbidities with clinically significant impairment of cardiac, renal, hepatic, or pulmonary function; iii) lacked complete follow-up data.

### Treatments

All patients underwent at least one cycle of platinum-based induction chemotherapy, while some also received additional concurrent or adjuvant therapies. The chemotherapy regimens included docetaxel-cisplatin-5-fluorouracil (TPF) (consisting of docetaxel 75 mg/m^2^, cisplatin 75 mg/m^2^ and 5-fluorouracil 750 mg/m^2^, every three weeks), docetaxel-cisplatin (TP) (consisting of docetaxel 75 mg/m^2^ and cisplatin 75 mg/m^2^, every three weeks), cisplatin-5-fluorouracil (PF) (5-fluorouracil 1000 mg/m^2^ and cisplatin 80mg/m^2^, every three weeks), and gemcitabine-cisplatin (GP) (gemcitabine 1,000 mg/m^2^, cisplatin 80mg/m^2^, every three weeks). All patients received induction chemotherapy followed by intensity-modulated radiation therapy (IMRT) with or without concurrent platinum-based chemotherapy. The gross tumor volume (GTV) consisted of the primary tumor (GTVnx) along with the metastatic lymph nodes (GTVnd). The clinical target volume (CTV) consisted of the high-risk clinical target volume (CTV1) as well as the low-risk clinical target volume (CTV2). The radiation doses were 68–76 Gy/31–33f, 60–70 Gy/31–33 f, 60–64 Gy/31–33 f, and 50–54 Gy/31–33 f, respectively. Although adverse effects such as myelosuppression and radiation-induced oral mucositis are unavoidable in patients receiving chemotherapy and radiotherapy, no patients required treatment interruptions or dose reductions due to severe adverse effects, nor were there any life-threatening complications.

### Tumor volume measurement

**Figure 1:**
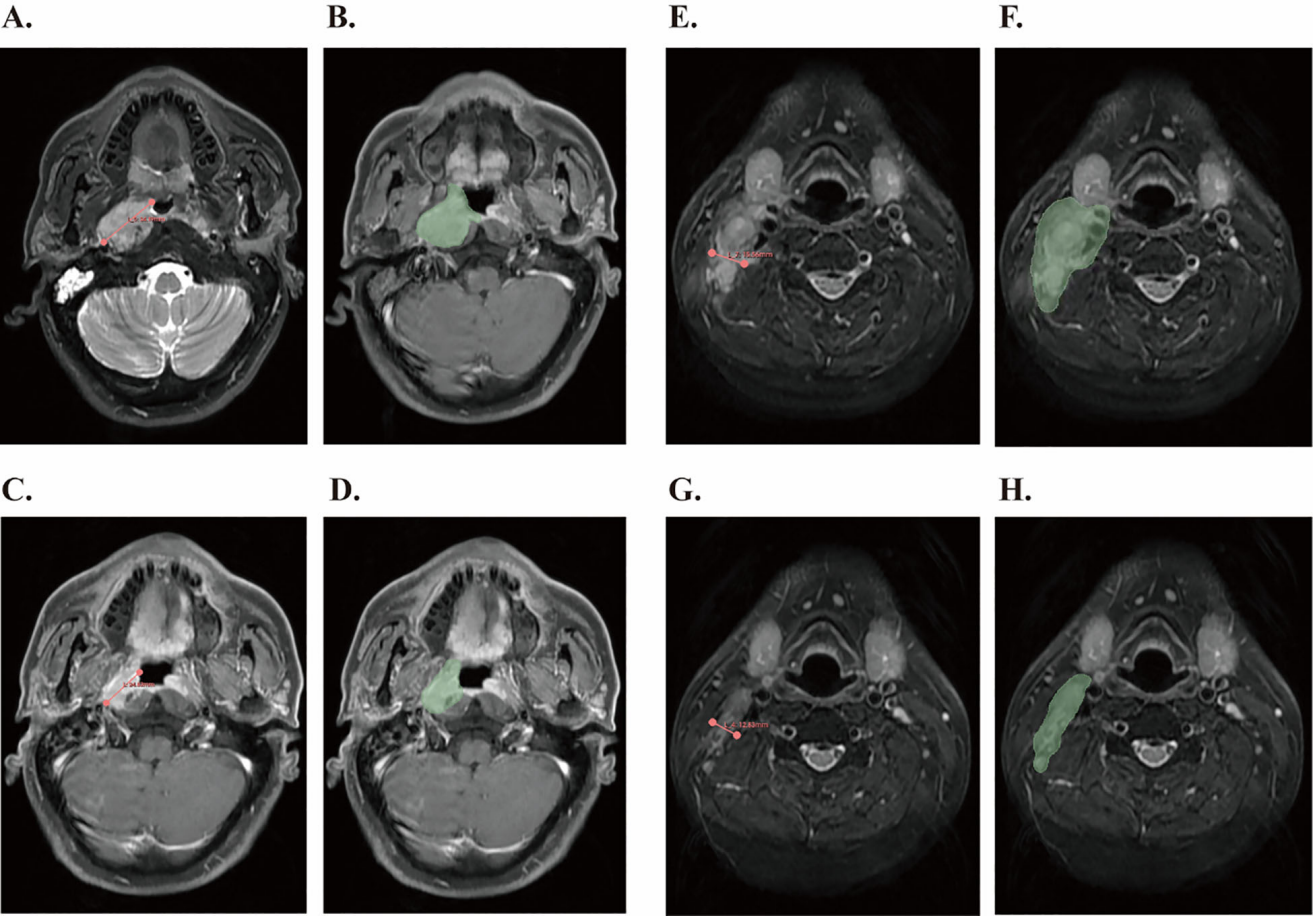
Tumor measurement using 3D Slicer software based on RECIST1.1 criteria and tumor volume segmentation before and after IC. **(A)** Measurement of the longest diameter of the primary lesion according to the RECIST1.1 standard before IC. **(B)** The primary gross tumor volume of the nasopharynx was contoured (green region) before IC. **(C)** Measurement of the shortest diameter of the cervical lymph nodes according to RECIST1.1 criteria after IC. **(D)** Metastatic cervical lymph node tumor volume was contoured (green region) after IC. **(E)** Measurement of the longest diameter of the primary lesion according to the RECIST1.1 standard before IC. **(F)** The primary gross tumor volume of the nasopharynx was contoured (green region) before IC. **(G)** Measurement of the shortest diameter of the cervical lymph nodes according to RECIST1.1 criteria after IC. **(H)** Metastatic cervical lymph node tumor volume was contoured (green region) after IC.

An example is presented in the **Figure 1**. Briefly, MRI images of patients were imported into the 3D SLICE software (a free open-source software platform). To ensure measurement accuracy and reproducibility, tumor assessments were independently performed by two trained investigators who were blinded to clinical outcomes. All measurements were subsequently reviewed and validated by a senior board-certified radiologist with over 15 years of experience in head and neck oncology imaging. In cases of discrepancy (>10% difference in lesion diameter), the final measurement was determined by consensus after joint re-evaluation on a dedicated workstation.

### Evaluation of RECIST 1.1 criteria

Each patient underwent at least two neck MRI examinations, one before and one after neoadjuvant chemotherapy. Two senior clinicians independently evaluated the resulting images according to RECIST version 1.1. According to these criteria, target lesions are defined as all measurable lesions, with a maximum of five lesions assessed and a maximum of two lesions were selected for each organ. Tumor lesions with a long diameter greater than 10mm were selected, and lymph node lesions with a short diameter greater than 15mm were selected. At baseline, the sum of the diameters of all target lesions (longest diameter for tumor lesions, shortest axis for the malignant lymph node) will be used as the basis for evaluation and comparison in the trial. A PR is defined as a reduction of at least 30% in the longest diameter of measurable target lesions.

### Follow-up and endpoints

All patients were consistently monitored until June 2024 or until they died and any recurrence or progression was documented. A structured follow-up schedule was implemented, with assessments every one to three months in the first two years, every six months for the next three to five years, and annually thereafter. The primary clinical endpoint was OS, which is the time from diagnosis until death or last follow-up. The secondary clinical endpoint was progression‐free survival (PFS) and distant metastasis-free survival (DMFS). PFS is defined as the time from the date of diagnosis to the date of disease progression (local, regional, or distant) or death from any cause, whichever occurs first. DMFS is defined as the time from the date of diagnosis to the date of the first occurrence of distant metastasis, and notably, patients with M1 and Mx stage at diagnosis were excluded from the DMFS analysis.

### Statistical analyses

This study utilized the software R (version 4.4.3), SPSS (version 25.0), and X-tile (version 3.6.1) for statistical analyses. Before statistical analysis, continuous variables were converted to categorical variables with the X-tile software. The Chi-square test assessed differences in clinicopathological characteristics between the two groups across the training set, internal validation set, and external validation set. After the univariate analysis, survival-associated variables were included in a multivariate Cox regression analysis. A predictive nomogram model was subsequently developed based on all candidate variables and was compared with the TNM staging system. The model’s discriminative ability was evaluated using the C-index and the area under the curve (AUC) of ROC. Calibration curves were utilized to assess the predictive accuracy of the model. DCA was employed to assess the clinical utility of the model. Individual risk scores were derived from the nomogram, and the truncated risk scores were calculated using the R software package to stratify patients by risk levels. Kaplan-Meier survival analysis was conducted to evaluate the statistical significance of OS incidence differences among the various risk groups.

## Results

### Characteristics of NPC patients from two institutions

Our study included a total of 561 eligible patients from two institutions (**Table 1**). From The First Affiliated Hospital of Guangxi Medical University, 504 patients diagnosed with stage II-IVB NPC were included. They were randomly assigned to a training cohort (n = 361) and an internal validation cohort (n = 143). Additionally, 57 patients with the same diagnosis from The First Affiliated Hospital of Hengyang Medical School, University of South China formed the external validation cohort (n = 57). The clinical and treatment characteristics are detailed in **Table 1**. The median age of the cohort was 47 years (range,13–68 years). Pathological examination indicated that most patients (n = 497, 98.5%) were classified as WHO grades II and III, which are the most common types found in endemic regions. The median follow-up duration for all patients was 63 months (range, 4–125 months). During this period, 57 (10.1%) patients experienced locoregional recurrence, 79 (14.1%) patients had distant recurrence, and 146 (26.0%) patients died.

**Table 1:** Basic information of included patients in this study.

Variables	Training cohort (n=361)	Internal validation cohort (n=143)	External validation cohort (n=57)	*P* value
Age				0.258
≤50y	132 (36.5%)	51 (35.6%)	24 (42.1%)	
<50	229 (63.5%)	92 (64.4%)	33 (57.9%)	
Sex				0.599
Male	266 (73.6%)	99 (69.3%)	41 (71.9%)	
Female	95 (26.4%)	44 (30.7%)	16 (28.1%)	
Pathology (WHO)				<0.001
I/III	5 (1.3%)	3 (2.1%)	15 (26.3%)	
II	356 (98.7%)	140 (97.9%)	42 (73.7%)	
Smoking				0.045
No	223 (61.7%)	84 (58.7%)	44 (77.2%)	
Yes	138 (38.3%)	59 (41.3%)	13 (22.8%)	
Drinking				0.446
No	268 (74.2%)	103 (72.0%)	46 (80.7%)	
Yes	93 (25.8%)	40 (28.0%)	11 (19.3%)	
T stage				0.040
T1	16 (4.4%)	11 (7.7%)	1 (1.8%)	
T2	48 (13.3%)	29 (20.3%)	9 (15.8%)	
T3	131 (36.3%)	40 (28.0%)	13 (22.8%)	
T4	166 (46.0%)	63 (44.0%)	34 (59.6%)	
N stage				0.060
N0	9 (2.5%)	2 (1.4%)	2 (3.5%)	
N1	89 (24.7%)	37 (25.9%)	7 (12.3%)	
N2	159 (44.0%)	59 (41.2%)	37 (64.9%)	
N3	104 (28.8%)	45 (31.5%)	11 (19.3%)	
M Stage	296 (82.0%)	118 (82.5%)	56 (98.2%)	
M0	296 (82.0%)	118 (82.5%)	56 (98.2%)	
M1	24 (6.6%)	13 (9.1%)	1 (1.8%)	
Mx	41 (11.4%)	12 (8.4%)	0 (0)	
Stage				0.497
II	14 (3.9%)	7 (4.9%)	1 (1.8%)	
III	110 (30.5%)	43 (30.1%)	16 (28.0%)	
IVA	212 (58.7%)	80 (50.9%)	39 (68.4%)	
IVB	25 (6.9%)	13 (9.1%)	1 (1.8%)	
IC cycle				0.194
≤2	327 (90.6%)	124 (86.7%)	54 (94.8%)	
>2	34 (9.4%)	19 (13.3%)	3 (5.2%)	
IC regimen				<0.001
TP	281 (77.8%)	114 (79.7^%)	40 (70.1%)	
PF	55 (15.3%)	18 (12.6%)	1 (1.8%)	
GP	0 (0)	1 (0.7%)	15 (26.3)	
TPF	25 (6.9%)	10 (7.0%)	1 (1.8%)	
Adjuvant chemotherapy				0.024
No	243 (67.3%)	96 (67.1%)	28 (49.1%)	
Yes	118 (32.7%)	47 (32.9%)	29 (50.9%)	
Induction platinum dosage				<0.001
<153.4	175 (48.5%)	71 (49.7%)	47 (82.5%)	
≥153.4	186 (51.5%)	72 (50.3%)	10 (17.5%)	
Concurrent platinum dosage				0.017
<201	315 (87.3%)	127 (88.8%)	57 (100%)	
≥201	46 (12.7%)	16 (11.2%)	0 (0%)	
Primary tumor volume after IC (mm^3^)				<0.001
<63.3	252 (69.8%)	104 (72.7%)	21 (36.8%)	
≥63.3	109 (30.2%)	39 (27.3%)	36 (63.2%)	
Cervical lymph node volume after IC (mm^3^)				0.516
<34.5	272 (75.3%)	108 (75.5%)	39 (68.4%)	
≥34.5	89 (24.7%)	35 (24.5%)	18 (31.6%)	
NLR				0.445
<2.7	213 (59.0%)	76 (53.1%)	31 (54.4%)	
≥2.7	148 (41.0%)	67 (46.9%)	26 (45.6%)	
PLR				0.022
<209	241 (66.8%)	79 (55.2%)	31 (54.4%)	
≥209	120 (33.2%)	64 (44.8%)	26 (45.6%)	
LMR				0.064
<2.1	92 (25.5%)	41 (28.7%)	23 (40.4%)	
≥2.1	269 (74.5%)	102 (71.3%)	34 (59.6%)	
EBV-DNA				0.555
Negative	103 (28.5%)	38 (26.6%)	23 (40.4)	
Positive	158 (43.8%)	68 (47.5%)	30 (52.6%)	
NA	100 (27.7%)	37 (25.9%)	4 (7%)	
PR rate				0.760
≤49%	169 (46.8%)	72 (50.3%)	28 (49.1%)	
>49%	192 (53.2%)	71 (49.7%)	29 (50.9%)	

IC, Induction chemotherapy; TP, Taxol + Cisplatin; PF, Cisplatin+5-Fluorouracil; GP, Gemcitabine + Cisplatin; TPF, Taxol+Cisplatin+5-Fluorouracil; NLR, Neutrophil to lymphocyte ratio; PLR, Platelet to lymphocyte ratio; LMR, Lymphocyte to monocyte ratio; PR, Partial remission; NA, Not available. Bold values indicate statistically significant results (*P* < 0.05).

### Establishment and validation of a nomogram model for OS

We performed univariate and multivariate analyses to identify independent prognostic factors related to OS, PFS and DMFS in the training cohort. The analyses revealed that age, M stage, primary tumor volume post-IC, cervical lymph nodes volume post-IC, LMR, and PR rate were significant independent prognostic factors for OS (**Table 2**). Similarly, these same factors were also identified as independent prognostic factors for PFS (**Supplementary Table S1**). For DMFS, however, the independent prognostic factors were found to be IC regimen, induction platinum dosage, and cervical lymph node volume after IC (**Supplementary Table S2**). These findings highlight the varying prognostic significance of different factors depending on the survival endpoint considered.

**Table 2:** Univariate and multivariate Cox regression analysis of clinical parameters for OS of NPC patients after induction chemotherapy in training cohort.

Variables	Univariate analysis		Multivariate analysis	
	HR (95% CI)	*P* value	HR (95% CI)	*P* value
Age (years)				
≤50	Reference		Reference	
>50	2.385 (1.378-4.130)	**0.002**	2.264 (1.287-3.982)	**0.005**
Sex				
Male	Reference			
Female	0.799 (0.485-1.316)	0.379		
Smoking				
No	Reference			
Yes	1.260 (0.825-1.926)	0.285		
Drinking				
No	Reference			
Yes	0.712 (0.419-1.210)	0.209		
Pathology (WHO)				
I	Reference			
II	1.205 (0.168-8.651)	0.853		
III	NA	NA		
T stage				
T1	Reference			
T2	0.666 (0.200-2.211)	0.506		
T3	0.582 (0.199-1.705)	0.324		
T4	1.378 (0.499-3.801)	0.536		
N stage				
N0	Reference		Reference	
N1	0.294 (0.096-0.903)	**0.032**	0.369 (0.116-1.176)	0.092
N2	0.472 (0.168-1.324)	0.154	0.520 (0.178-1.518)	0.231
N3	0.722 (0.256-2.040)	0.539	0.789 (0.259-2.406)	0.677
M stage				
M0	Reference		Reference	
M1	2.528 (1.336-4.786)	**0.004**	2.210 (1.120-4.358)	**0.022**
Mx	1.187 (0.609-2.310)	0.615	1.231 (0.618-2.452)	0.555
Stage				
II	Reference			
III	0.516 (0.149-1.786)	0.296		
IVA	1.143 (0.358-3.654)	0.821		
IVB	2.112 (0.587-7.597)	0.252		
IC cycle				
≤2	Reference			
>2	1.098 (0.529-2.281)	0.801		
IC regimen				
TP	Reference			
PF	1.626 (0.981-2.696)	0.059		
GP	NA	NA		
TPF	1.259 (0.542-2.925)	0.592		
Adjuvant chemotherapy				
No	Reference			
Yes	0.975 (0.863-1.101)	0.728		
Induction platinum dosage				
<153.4	Reference		Reference	
≥153.4	1.872 (1.208-2.900)	**0.005**	1.453 (0.921-2.293)	0.108
Concurrent platinum dosage				
<201	Reference		Reference	
≥201	1.814 (1.084-3.126)	**0.024**	1.184 (0.669-2.097)	0.562
Primary tumor volume after IC (mm^3^)				
<63.3	Reference		Reference	
≥63.3	3.414 (2.242-5.198)	**<0.001**	3.295 (2.103-5.162)	**<0.001**
Cervical lymph node volume after IC (mm^3^)				
<34.5	Reference		Reference	
≥34.5	2.354 (1.531-3.618)	**<0.001**	2.159 (1.338-3.485)	**0.002**
NLR				
<2.7	Reference			
≥2.7	1.132 (0.742-1.726)	0.564		
PLR				
≥209	Reference		Reference	
≥209	1.560 (1.021-2.382)	**0.040**	0.868 (0.507-1.488)	0.607
LMR				
<2.1	Reference		Reference	
≥2.1	0.527 (0.342-0.812)	**0.004**	0.561 (0.326-0.965)	**0.037**
EBV-DNA				
Negative	Reference			
Positive	0.728 (0.456-1.163)	0.184		
PR rate				
≤49%	Reference		Reference	
>49%	0.507 (0.330-0.778)	**0.002**	0.606 (0.389-0.945)	**0.027**

IC, Induction chemotherapy; TP, Taxol + Cisplatin; PF, Cisplatin+5-Fluorouracil; GP, Gemcitabine + Cisplatin; TPF, Taxol + Cisplatin + 5-Fluorouracil; NLR, Neutrophil to lymphocyte ratio; PLR, Platelet to lymphocyte ratio; LMR, Lymphocyte to monocyte ratio; PR, Partial remission ratio; NA, Not available. Bold values indicate statistically significant results (*P* < 0.05).

**Figure 2:**
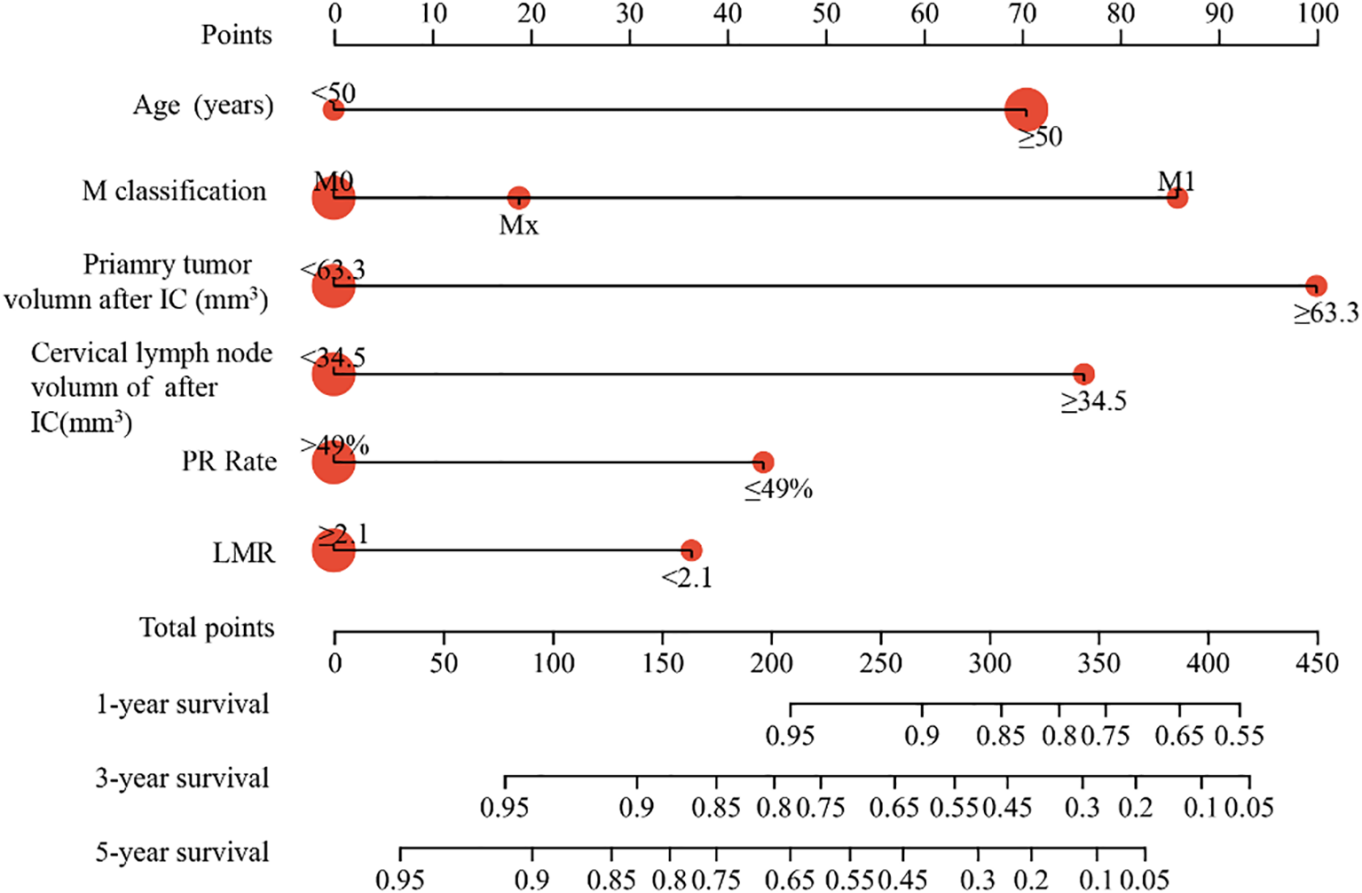
Construction of nomogram in the training cohort to predict the OS of NPC patients. The nomogram model predicted the 1-, 3- and 5-years OS of NPC patients in training cohort.

**Figure 3:**
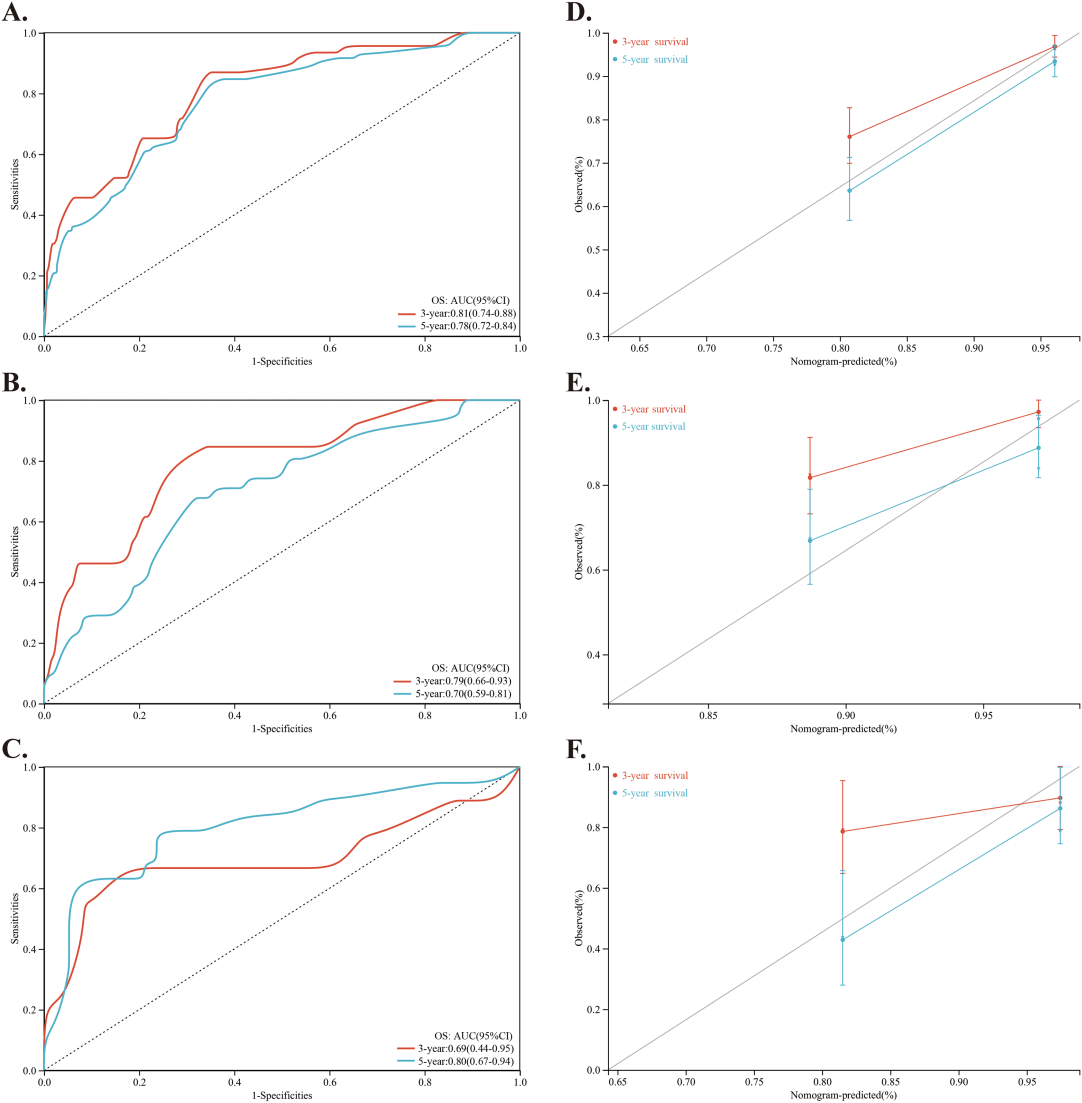
Investigation and validation for the predictive role of the nomogram. **(A–C)** Time-dependent ROC curves of the nomogram for predicting 3-year and 5-year OS of NPC patients in the training cohort, internal validation cohort and external validation cohort, respectively. **(D–F)** Calibration curve of the nomogram for predicting 3-year and 5-year OS of NPC patients in the training cohort, internal validation cohort and external validation cohort, respectively.

Using these factors, we created a nomogram in the training cohort to predict the probabilities of 1-year, 3-year and 5-year OS (**Figure 2**). We calculated the C-index to be 0.769 (95% CI: 0.718-0.819). In the training cohort, the AUC for our predictive model were 0.81 for 3-year OS and 0.78 for 5-year OS (**Figure 3A**). In the internal validation cohort, the AUC for our prediction model regarding 3-year and 5-year OS were 0.79 and 0.70, respectively (**Figure 3B**). We also validated the models with an external cohort, which showed AUCs of 0.69 for 3-year OS and 0.80 for 5-year OS (**Figure 3C**). Additionally, to verify the stability and generalization ability of the nomogram, we further used the 5-fold cross-validation method in the original cohort from The First Affiliated Hospital of Guangxi Medical University. The AUC for the model predicting 3-year and 5-year OS were 0.75 and 0.66, respectively (**Supplementary Figure S1**). Furthermore, across all cohorts, the calibration curve of the model demonstrated a strong concordance between observed outcomes and predicted probabilities, indicating that the model exhibits good sensitivity and specificity (**Figures 3D-F**).

### Comparison of predictive accuracy between nomogram and TNM staging system

**Figure 4:**
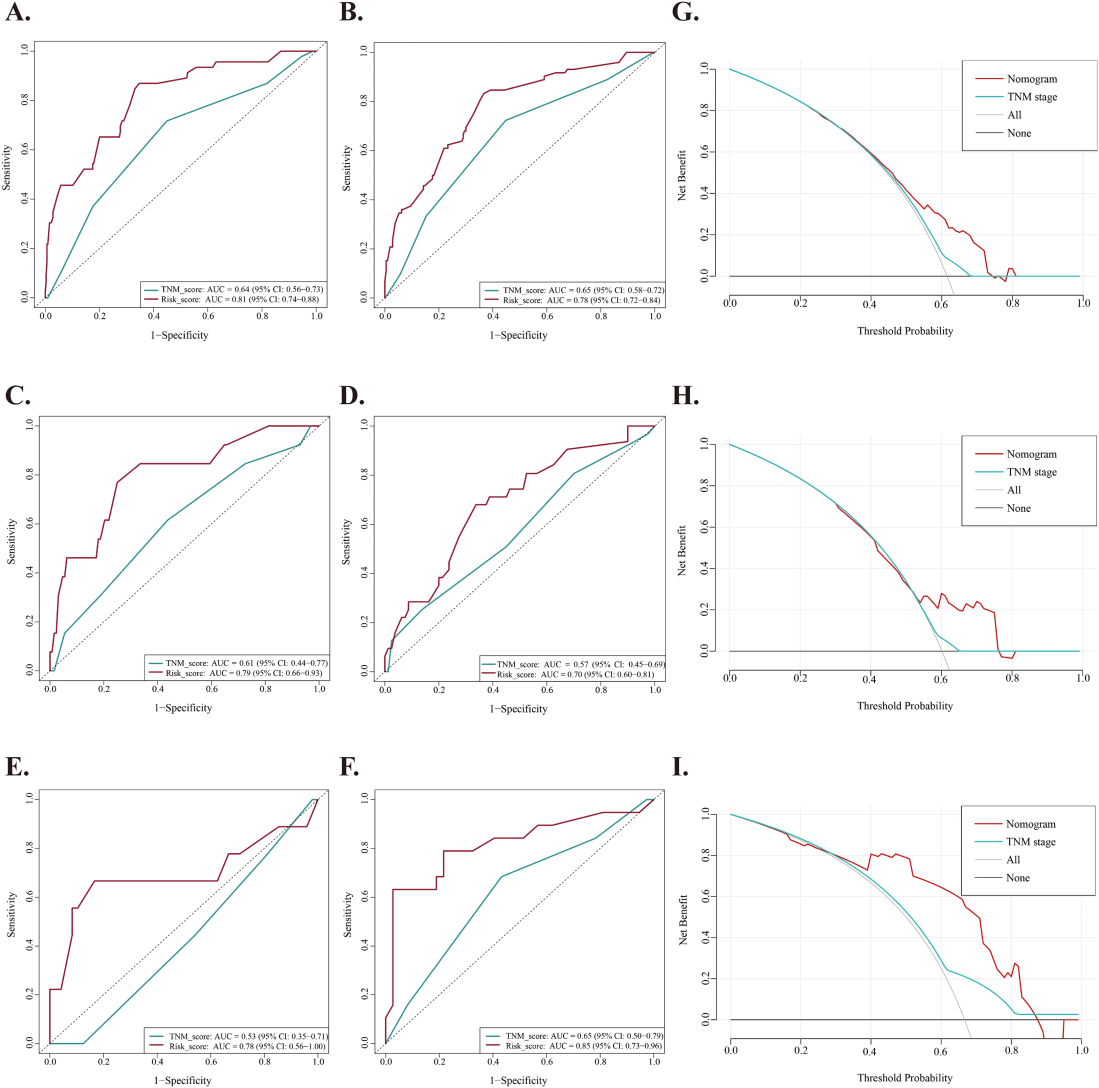
Comparison of predictive accuracy between nomogram and TNM staging system. **(A, B)** Time-dependent ROC curves of the nomogram and TNM staging system for predicting 3-year and 5-year OS of NPC patients in the training cohort, respectively. **(C, D)** Time-dependent ROC curves of the nomogram and TNM staging system for predicting 3-year and 5-year OS of NPC patients in the internal validation cohort, respectively. **(E, F)** Time-dependent ROC curves of the nomogram and TNM staging system for predicting 3-year and 5-year OS of NPC patients in the external validation cohort, respectively. **(G–I)** Decision curves analysis (DCA) of the nomogram and TNM staging system for predicting OS of NPC patients in the training cohort, internal validation cohort and external validation cohort, respectively.

We evaluated the predictive accuracy of the proposed nomogram compared to the 8th edition TNM staging system. The time-dependent ROC analysis showed that the nomogram had superior predictive accuracy for OS in the training cohort, with 3-year AUCs of 0.81 compared to 0.64, and 5-year AUCs of 0.78 versus 0.65 (**Figures 4A, B**). The C-index of these two models for outcome prediction was 0.769 (95%CI: 0.715-0.819) versus 0.661 (95%CI:0.588-0.735). Similarly, in the internal validation cohort, the nomogram achieved a 3-year AUC of 0.79 versus 0.61, and a 5-year AUC of 0.70 versus 0.59 (**Figures 4C, D**). The C-index was 0.713 (95%CI: 0.615-0.805) versus 0.581 (95% CI 0.455-0.702). In the external validation cohort, the nomogram achieved a 3-year AUC of 0.78 compared to 0.53, and a 5-year AUC of 0.85 versus 0.65 (**Figures 4E, F**). The C-index was 0.762 (95%CI: 0.629-0.884) versus 0.606 (95% CI 0.441-0.771). These analyses indicated that the new nomogram provides better clinical discrimination. In addition, the DCA showed that the nomogram provided more accurate predictions of 3- and 5-year OS across a wider risk threshold interval than the TNM staging system, as illustrated in **Figures 4G-I**.

### Nomogram score for risk stratification

**Figure 5:**
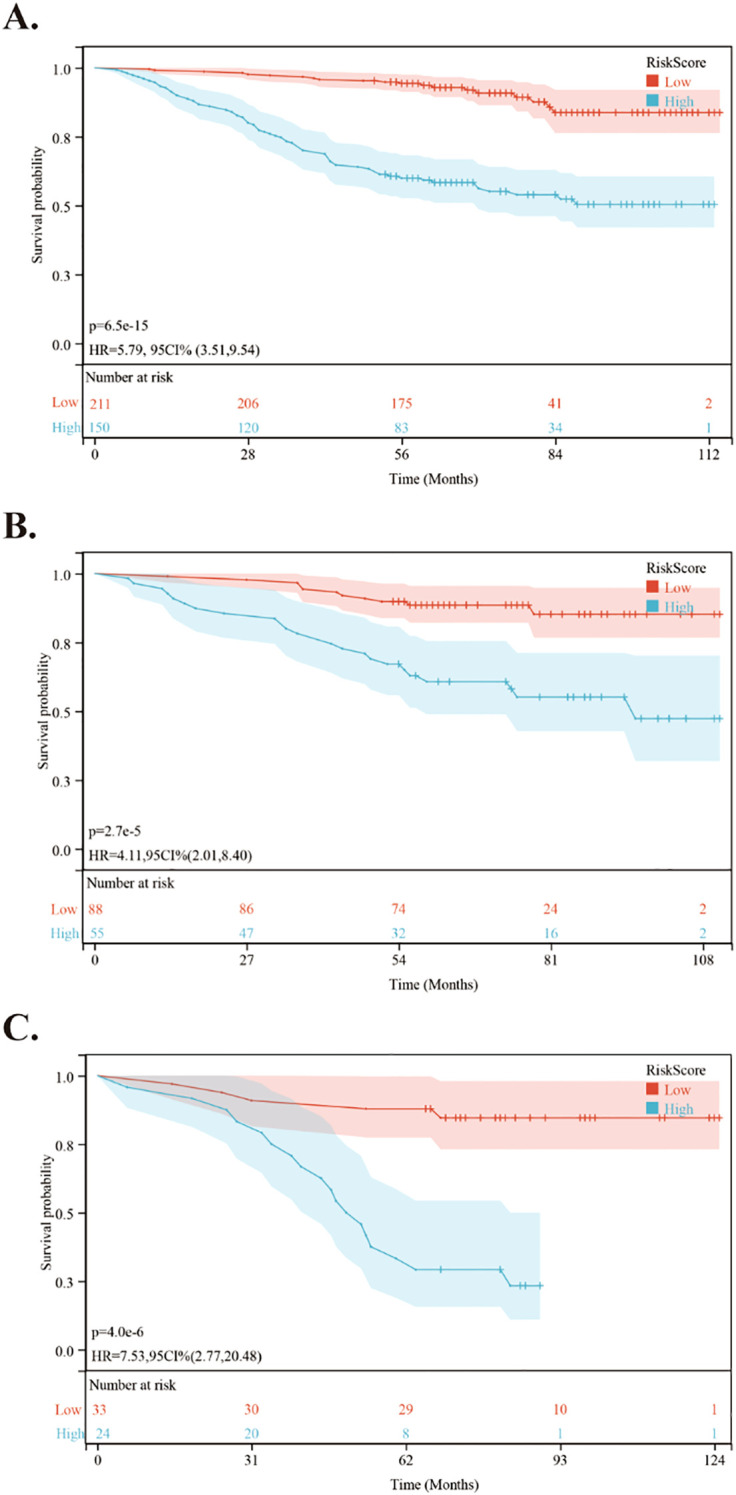
Kaplan-Meier survival curves comparing OS between low- and high-risk groups in the training and validation cohorts. **(A)** Kaplan-Meier survival curves comparing OS between low- and high-risk groups in the training cohort. **(B)** Kaplan-Meier survival curves comparing OS between low- and high-risk groups in the internal validation cohort. **(C)** Kaplan-Meier survival curves comparing OS between low- and high-risk groups in the external validation cohort.

**Figure 6:**
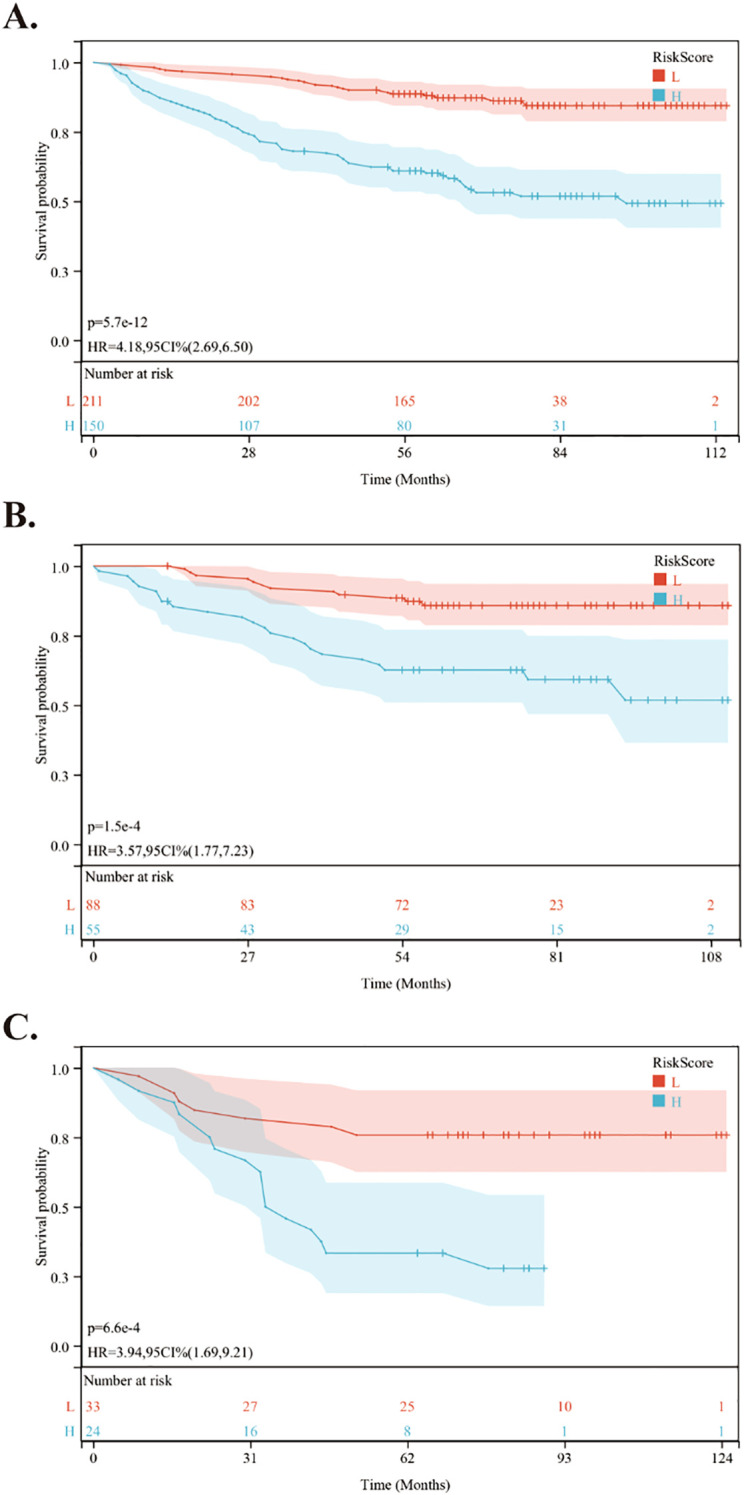
Kaplan-Meier survival curves comparing PFS between low- and high-risk groups in the training and validation cohorts. **(A)** Kaplan-Meier survival curves comparing PFS between low- and high-risk groups in the training cohort. **(B)** Kaplan-Meier survival curves comparing PFS between low- and high-risk groups in the internal validation cohort. **(C)** Kaplan-Meier survival curves comparing PFS between low- and high-risk groups in the external validation cohort.

**Figure 7:**
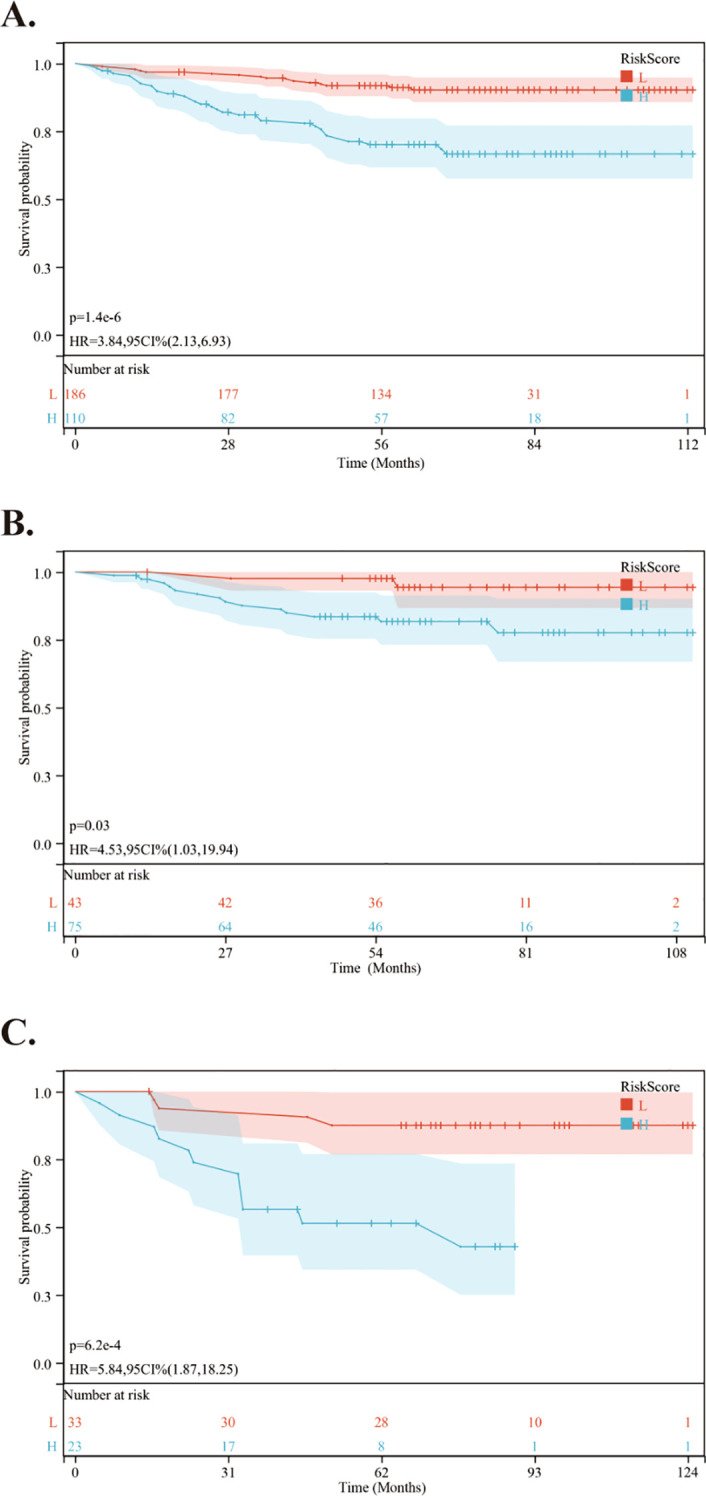
Kaplan-Meier survival curves comparing DMFS between low- and high-risk groups in the training and validation cohorts. **(A)** Kaplan-Meier survival curves comparing DMFS between low- and high-risk groups in the training cohort. **(B)** Kaplan-Meier survival curves comparing DMFS between low- and high-risk groups in the internal validation cohort. **(C)** Kaplan-Meier survival curves comparing DMFS between low- and high-risk groups in the external validation cohort.

Using the nomogram, we calculated the risk scores for all patients. The “maxstat” R package was employed to find the optimal cutoff value for the risk score. This value was determined to be 0.0905. Based on this cutoff value, patients were divided into high-risk and low-risk groups. We then conducted further analyses to assess the prognosis and survival outcomes of patients in each group. Statistically significant difference in OS (**Figure 5**), PFS (**Figure 6**) and DMFS (**Figure 7**) was observed among these groups (*p* < 0.001).

### Subgroup analyses

We conducted a subgroup analysis of patients from the First Affiliated Hospital of Guangxi Medical University based on the PR rate. In the PR ≤ 0.49 group: 3-year OS: 84.6%, 3-year PFS: 88.7%, 3-year DMFS: 85.2%; 5-year OS: 69.8%, 5-year PFS: 83.4%, 5-year DMFS: 78.8%. In the PR > 0.49 group: 3-year OS: 93.9%, 3-year PFS: 96.4%, 3-year DMFS: 93.5%; 5-year OS: 87.2%, 5-year PFS: 93.3%, 5-year DMFS: 89.4%. Furthermore, we conducted the univariate and multivariate Cox regression analyses for OS of NPC patients. As presented in **Table 3**, the univariate Cox regression analyses indicated that smoking status, cancer stage, cumulative dose of concurrent chemotherapy with cisplatin, tumor volume post-IC, and platelet-to-lymphocyte ratio (PLR) were significantly associated with OS in the group with a PR rate of ≤ 49%. However, multivariate analysis identified only the cumulative dose of concurrent chemotherapy with cisplatin as an independent prognostic factor for OS (HR, 2.04; 95%CI, 1.118-3.721; *p* = 0.02). In the group with a PR rate greater than 49%, cervical lymph nodes volume following induction chemotherapy (HR = 4.577; 95%CI = 2.454-8.536; p < 0.001) and the LMR (HR = 0.486; 95% CI = 0.252-0.934; p = 0.03) were identified as independent prognostic factors for OS through both univariate and multivariate analyses.

**Table 3:** Univariate and multivariate Cox regression analyses for OS of NPC patients with different PR rate.

Variables	Group 1 (PR rate ≤49%)				Group 2 (PR rate >49%)			
	Univariate analysis		Multivariate analysis		Univariate analysis		Multivariate analysis	
	HR (95% CI)	*P* value	HR (95% CI)	*P* value	HR (95% CI)	*P* value	HR (95% CI)	*P* value
Age (years)								
≤50	Reference				Reference			
>50	1.170 (0.666-2.055)	0.585			1.902 (0.893-4.047)	0.100		
Sex								
Male	Reference				Reference			
Female	0.795 (0.483-1.311)	0.369			0.847 (0.418-1.714)	0.644		
Smoking								
No	Reference		Reference		Reference			
Yes	1.647 (1.057-2.568)	**0.028**	1.317 (0.784-2.222)	0.298	0.867 (0.469-1.603)	0.649		
Drinking								
No	Reference				Reference			
Yes	1.069 (0.637-1.794)	0.800			0.939 (0.473-1.866)	0.858		
Pathology (WHO)								
I	Reference				Reference			
II	0.996 (0.138-7.171)	0.997			0.536 (0.084-3.429)	0.510		
III	NA	NA			NA	NA		
T stage								
T1	Reference		Reference		Reference			
T2	0.342 (0.122-0.963)	**0.042**	0.395 (0.121-1.294)	0.125	3.237 (0.398-26.334)	0.272		
T3	0.293 (0.114-0.751)	**0.011**	0.375 (0.130-1.083)	0.070	2.523 (0.332-19.193)	0.371		
T4	0.636 (0.272-1.489)	0.297	0.500 (0.171-1.463)	0.206	3.291 (0.444-24.421)	0.244		
N stage								
N0	Reference				Reference			
N1	0.396 (0.129-1.216)	0.106			0.370 (0.045-3.014)	0.350		
N2	0.613 (0.217-1.732)	0.356			0.473 (0.063-3.560)	0.470		
N3	0.786(0.276-2.237)	0.652			1.043 (0.139-7.808)	0.970		
M stage								
M0	Reference				Reference			
M1	1.134 (0.807-1.594)	0.468			1.445 (0.997-2.095)	0.052		
Mx	0.939 (0.406-2.175)	0.884			1.799 (0.791-4.090)	0.160		
Stage								
II	Reference				Reference			
III	1.288 (0.294-5.634)	0.737			0.400 (0.087-1.838)	0.239		
IVA	2.039 (0.496-8.374)	0.323			0.666 (0.157-2.826)	0.582		
IVB	3.893 (0.862-17.572)	0.077			1.445 (0.289-7.221)	0.654		
IC cycle								
≤2	Reference				Reference			
>2	0.803 (0.386-1.672)	0.558			0.943 (0.759-4.974)	0.166		
IC regimen								
TP	Reference		Reference		Reference			
PF	1.983 (1.200-3.277)	**0.008**	1.732 (0.993-3.020)	0.053	1.924 (0.262-14.125)	0.520		
GP	NA	NA			NA	NA		
TPF	1.353 (0.611-2.994)	0.456	1.530 (0.359-6.516)	0.565	2.813 (0.347-22.791)	0.330		
Adjuvant chemotherapy								
No	Reference				Reference	0.817		
Yes	0.975 (0.863-1.101)	0.681			0.969 (0.742-1.265)			
Induction platinum dosage								
<153.4	Reference				Reference	0.094		
≥153.4	1.530 (0.976-2.400)	0.064			1.691 (0.914-3.126)			
Concurrent platinum dosage								
<201	Reference		Reference		Reference	0.581		
≥201	2.032 (1.140-3.623)	**0.016**	2.040 (1.118-3.721)	**0.020**	1.255 (0.559-2.816)			
Primary tumor volume after IC (mm^3^)								
<63.3	Reference		Reference		Reference	0.120		
≥63.3	2.078 (1.288-3.353)	**0.003**	1.582 (0.951-2.632)	0.077	1.654 (0.871-3.142)			
Cervical lymph node volume after IC (mm^3^)								
<34.5	Reference				Reference		Reference	
≥34.5	1.429 (0.870-2.346)	0.158			4.109 (2.235-7.553)	**<0.001**	4.577 (2.454-8.536)	**<0.001**
NLR								
<2.7	Reference				Reference	0.430		
≥2.7	1.145 (0.735-1.785)	0.549			1.269 (0.699-2.306)			
PLR								
<209	Reference		Reference		Reference	0.600		
≥209	1.604 (1.028-2.503)	**0.037**	1.344 (0.823-2.195)	0.238	1.184 (0.635-2.209)			
LMR								
<2.1	Reference				Reference		Reference	
≥2.1	0.651 (0.411–1.031)	0.068			0.540 (0.292-0.999)	**0.050**	0.486 (0.252-0.934)	**0.030**
EBV-DNA								
Negative	Reference				Reference			
Positive	0.942 (0.576-1.541)	0.811			1.308 (0.648-2.644)	0.450		

IC, Induction chemotherapy; TP, Taxol + Cisplatin; PF, Cisplatin+5-Fluorouracil; GP, Gemcitabine + Cisplatin; TPF, Taxol + Cisplatin + 5-Fluorouracil; NLR, Neutrophil to lymphocyte ratio; PLR, Platelet to lymphocyte ratio; LMR, Lymphocyte to monocyte ratio; PR, Partial remission ratio; NA, Not available. Bold values indicate statistically significant results (*P* < 0.05).

## Discussion

In this study, we aimed to evaluate the clinical significance of tumor responses at different rates of progression following induction chemotherapy. To achieve this, we developed a nomogram incorporating these variables to aid in predicting the prognosis of NPC post-IC. Since not all patients respond favorably to induction chemotherapy, and since multiple studies have linked different tumor responses after chemotherapy to patient prognosis, it is especially important to investigate the progression rate. This study presents the first validated nomogram that integrates different rates of progression, TNM staging systems, serum biomarkers, and MRI-derived tumor characteristics to predict OS in patients with NPC. Furthermore, the prognostic accuracy of the nomogram exceeds that of the eighth edition of the TNM staging system. Subsequently, patients were effectively stratified into high-risk and low-risk groups, demonstrating a significant difference in 5-year OS. These findings indicate that different rates of tumor progression are significant for the prognosis of NPC patients undergoing induction chemotherapy and can help clinicians make informed treatment decisions.

Numerous studies have shown that different tumor responses following IC correlate with prognosis (18–20). Previous studies explored and found that volumetric reductions of target lesion after IC are independent survival predictor, and outperformed unidimensional measurement and RECIST guideline for NPC patients (21–23). Another retrospective study analyzed patients treated with intensity-modulated radiation therapy (IMRT) and IC, finding that tumor response to IC is an independent prognostic factor for disease-free survival (DFS), OS, and locoregional relapse-free survival (LRRFS). CR was observed in 101 out of 399 patients (25.3%), PR in 262 patients (65.7%), and SD in 36 patients (9.0%) (20). A study using a tumor response nomogram also found it to be a significant predictor of OS in patients with locally advanced NPC. Of the patients, 340 (68.3%) demonstrated treatment efficacy classified as CR or PR. The survival outcomes for patients with CR/PR were superior to those with SD or PD (24). Nonetheless, no research has yet established a correlation between the PR remission rate and OS in cases of nasopharyngeal carcinoma. A substantial proportion of patients achieved PR, defined by the RECIST 1.1 criteria as a reduction in the target lesion’s maximum diameter by at least 30% for at least four weeks. Consequently, it is imperative to investigate patients whose tumor shrinkage rate following induction chemotherapy ranges between 30% and 100%.

Currently, many prognostic indicators for patients with NPC have been identified, alongside the extensively used TNM staging system. Age is a well-established risk factor for NPC. Evidence indicates that older patients tend to have a worse prognosis than younger patients (25, 26), although specific age thresholds differ among studies. In our study, we categorize nasopharyngeal cancer patients into early-onset and late-onset groups, using 50 years as the cutoff age. The findings indicated that patients with late-onset disease exhibited a poorer prognosis (27). This observation was corroborated within the cohort of patients undergoing induction chemotherapy in this study. The poorer prognosis in elderly patients may result from their reduced treatment tolerance and differences in tumor heterogeneity (28). The TNM stage emerged as a significant prognostic factor, a conclusion supported by our multifactorial analysis. Typically, the TNM stage is determined based on the size and/or extent of the primary tumor and/or metastatic lymph nodes. However, it occasionally fails to accurately represent the actual tumor burden (29). Single or two-dimensional measurements are important, but three-dimensional volumetric assessment is increasingly crucial, especially for non-surgical treatments like radiotherapy or chemotherapy. Numerous studies have highlighted the prognostic value of tumor volume across various cancers, with increasing evidence showing that both pre-treatment tumor volume and residual volume are prognostically significant (30–32). Our research further demonstrated the prognostic implications of residual volume following induction chemotherapy from a three-dimensional perspective. Recent studies have shown that the systemic inflammatory response is a key factor in tumor progression and prognosis. Earlier studies have demonstrated that inflammatory cells release cytokines into the tumor microenvironment, thereby facilitating tumor growth, angiogenesis, invasion, and metastasis. In cancer prognosis, inflammation-related markers have garnered significant scholarly attention. The LMR is a critical component of the immune response during inflammation. It has been extensively studied and recognized as a significant prognostic indicator in various solid tumors, including NPC, pancreatic cancer, and especially lung cancer. For example, Chan et al. identified LMR as an independent prognostic factor in a study of 1,623 colorectal cancer (CRC) patients undergoing curative resection (33). Our study substantiates this conclusion, reinforcing the role of LMR as a dependable prognostic marker in CRC.

In the subgroup analysis, patients with a PR rate below 49% benefited from cumulative cisplatin doses given during concurrent chemoradiotherapy. Previous investigations by Tang et al. and Wen et al. examined the prognostic implications of cumulative cisplatin dosage (34, 35), corroborating our findings. While the cumulative cisplatin dose did not exhibit a significant association with survival outcomes across the entire cohort, a notable correlation was observed in patients with a PR rate below 49%. In contrast, for patients with a higher PR rate, increasing the cumulative cisplatin dose did not improve their prognosis. This suggests that a standard cumulative dose of cisplatin may not be required for all NPC patients, which is crucial for tailoring future treatment strategies, particularly for those undergoing induction chemotherapy.

Currently, the treatment of NPC mainly depends on TNM staging. However, because of tumor heterogeneity, patients at the same stage can show significant differences in their prognosis. Our study developed a nomogram that includes the PR rates after induction chemotherapy, which helps identify patients likely to benefit from this treatment. This model offers valuable guidance for clinical practice by assisting oncologists in choosing personalized treatment strategies for their patients. Further investigation is needed, even though the nomogram accurately predicts OS. The study does have certain limitations. First, as a retrospective analysis, it may be subject to selection bias. A prospective study with multiple centers to validate the adaptability and generalizability of the model is needed in future. Additionally, although EBV DNA levels were included, the significant amount of missing data could potentially introduce bias in the results.

## Conclusions

In conclusion, the rates of PR following post-induction chemotherapy are significantly associated with overall survival outcomes in patients with NPC. Furthermore, a nomogram that incorporates PR rates along with other variables shows improved predictive ability for OS compared to the current TNM staging system, thus equipping clinicians with a more precise tool for guiding treatment strategies. Additional prospective studies are essential to validate these findings and facilitate their integration into clinical practice.

## Data Availability

The raw data supporting the conclusions of this article will be made available by the authors, without undue reservation.
