# Supplementary material for: Establishment and validation of a prognostic model for nasopharyngeal carcinoma patients based on partial response rates
Source: Front Oncol. 2025 Nov 19;15:1705634. doi: 10.3389/fonc.2025.1705634 (PMC12672269; doi:10.3389/fonc.2025.1705634)
Supplement: Supplementary file 1 [file DataSheet1.docx]

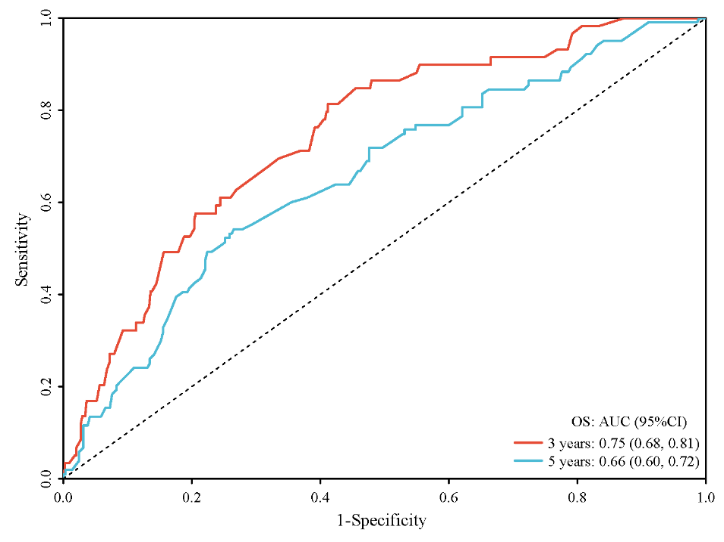


**Figure S1. Investigation of the predictive role for the nomogram using the cross-validation method.**

Five-fold cross-validation method was performed to verify the stability and generalization ability of the nomogram in the original cohort from The First Affiliated Hospital of Guangxi Medical University.
