# Supplementary material for: Establishment and validation of a prognostic model for nasopharyngeal carcinoma patients based on partial response rates
Source: Front Oncol. 2025 Nov 19;15:1705634. doi: 10.3389/fonc.2025.1705634 (PMC12672269; doi:10.3389/fonc.2025.1705634)
Supplement: Supplementary file 2 [file Table1.docx]

| **Table S1. Univariate and multivariate Cox regression analysis of clinical parameters for PFS of**  **NPC patients after induction chemotherapy in training cohort** | | | | | |
| --- | --- | --- | --- | --- | --- |
|  | Univariate analysis | | Multivariate analysis | |  |
| Variables | HR (95% CI) | *p* value | HR (95% CI) | *p* value |  |
| Age |  |  |  |  |  |
| ≤50 | Reference |  | Reference |  |  |
| >50 | 1.667 (1.043-2.663) | **0.033** | 1.582 (1.181-2.550) | **0.048** |  |
| Sex |  |  |  |  |  |
| Male | Reference |  |  |  |  |
| Female | 0.596 (0.352-1.007) | 0.053 |  |  |  |
| Smoking |  |  |  |  |  |
| No | Reference |  |  |  |  |
| Yes | 1.330 (0.884-2.001) | 0.171 |  |  |  |
| Drinking |  |  |  |  |  |
| No | Reference |  |  |  |  |
| Yes | 1.062 (0.671-1.680) | 0.797 |  |  |  |
| Pathology (WHO) |  |  |  |  |  |
| I | Reference |  |  |  |  |
| II | 1.317 (0.184-9.449) | 0.784 |  |  |  |
| III | NA | NA |  |  |  |
| T stage |  |  |  |  |  |
| T1 | Reference |  |  |  |  |
| T2 | 0.383 (0.129-1.140) | 0.085 |  |  |  |
| T3 | 0.445 (0.181-1.094) | 0.078 |  |  |  |
| T4 | 0.985 (0.425-2.285) | 0.973 |  |  |  |
| N stage |  |  |  |  |  |
| N0 | Reference |  |  |  |  |
| N1 | 0.404 (0.136-1.203) | 0.103 |  |  |  |
| N2 | 0.502 (0.179-1.408) | 0.190 |  |  |  |
| N3 | 0.833 (0.296-2.343) | 0.729 |  |  |  |
| M stage |  |  |  |  |  |
| M0 | Reference |  | Reference |  |  |
| M1 | 2.357 (1.249-4.448) | **0.008** | 2.387 (1.243-4.585) | **0.022** |  |
| Mx | 1.166 (0.618-2.202) | 0.615 | 1.221 (0.640-2.329) | 0.544 |  |
| Stage |  |  |  |  |  |
| II | Reference |  |  |  |  |
| III | 0.594 (0.174-2.030) | 0.406 |  |  |  |
| IVA | 1.245 (0.390-3.970) | 0.711 |  |  |  |
| IVB | 2.401 (0.677-8.523) | 0.175 |  |  |  |
| IC cycle |  |  |  |  |  |
| ≤2 | Reference |  |  |  |  |
| >2 | 1.270 (0.657-2.453) | 0.478 |  |  |  |
| IC regimen |  |  |  |  |  |
| TP | Reference |  |  |  |  |
| PF | 1.568 (0.950-2.586) | 0.078 |  |  |  |
| GP | NA | NA |  |  |  |
| TPF | 1.332 (0.609-2.912) | 0.473 |  |  |  |
| Adjuvant chemotherapy |  |  |  |  |  |
| No | Reference |  |  |  |  |
| Yes | 1.020 (0.663-1.568) | 0.928 |  |  |  |
| Induction platinum dosage |  |  |  |  |  |
| <153.4 | Reference |  | Reference |  |  |
| ≥153.4 | 1.740 (1.145-2.644) | **0.010** | 1.496 (0.979-2.286) | 0.063 |  |
| Concurrent platinum dosage |  |  |  |  |  |
| <201 | Reference |  |  |  |  |
| ≥201 | 1.590 (0.928-2.724) | 0.091 |  |  |  |
| Primary tumor volume after IC (mm^3^) |  |  |  |  |  |
| <63.3 | Reference |  | Reference |  |  |
| ≥63.3 | 2.505 (1.670-3.759) | **<0.001** | 2.287 (1.513-3.458) | **<0.001** |  |
| Cervical lymph node volume after IC (mm^3^) |  |  |  |  |  |
| <34.5 | Reference |  | Reference |  |  |
| ≥34.5 | 1.905 (1.243-2.920) | **0.003** | 1.979 (1.283-3.053) | **0.002** |  |
| NLR |  |  |  |  |  |
| <2.7 | Reference |  |  |  |  |
| ≥2.7 | 1.087 (0.722-1.636) | 0.689 |  |  |  |
| PLR |  |  |  |  |  |
| <209 | Reference |  |  |  |  |
| ≥209 | 1.471 (0.974-2.210) | 0.066 |  |  |  |
| LMR |  |  |  |  |  |
| <2.1 | Reference |  | Reference |  |  |
| ≥2.1 | 0.547 (0.359-0.833) | **0.005** | 0.646 (0.420-0.994) | **0.047** |  |
| EBV-DNA |  |  |  |  |  |
| Negative | Reference |  |  |  |  |
| Positive | 0.744 (0.475-1.165) | 0.197 |  |  |  |
| PR rate |  |  |  |  |  |
| ≤49% | Reference |  | Reference |  |  |
| >49% | 0.546 (0.362-0.825) | **0.004** | 0.637 (0.420-0.968) | **0.034** |  |
| IC: Induction chemotherapy; TP: Taxol + Cisplatin; PF: Cisplatin+5-Fluorouracil; GP: Gemcitabine + Cisplatin; TPF: Taxol + Cisplatin + 5-Fluorouracil; NLR: Neutrophil to lymphocyte ratio; PLR: Platelet to lymphocyte ratio; LMR: Lymphocyte to monocyte ratio; PR: Partial remission ratio; NA: Not available | | | | |  |

| **Table S2. Univariate and multivariate Cox regression analysis of clinical parameters for DMFS of**  **NPC patients after induction chemotherapy in training cohort** | | | | | | |
| --- | --- | --- | --- | --- | --- | --- |
|  | Univariate analysis | | Multivariate analysis | | |  |
| Variables | HR (95% CI) | *p* value | HR (95% CI) | *p* value | |  |
| Age |  |  |  |  |  |  |
| ≤50 | Reference |  |  |  | |  |
| >50 | 1.605 (0.849-3.037) | 0.145 |  |  | |  |
| Sex |  |  |  |  | |  |
| Male | Reference |  |  |  | |  |
| Female | 0.675 (0.337-1.352) | 0.267 |  |  | |  |
| Smoking |  |  |  |  | |  |
| No | Reference |  |  |  | |  |
| Yes | 0.991 (0.558-1.761) | 0.976 |  |  | |  |
| Drinking |  |  |  |  | |  |
| No | Reference |  |  |  | |  |
| Yes | 0.910 (0.474-1.746) | 0.777 |  |  | |  |
| Pathology (WHO) |  |  |  |  | |  |
| I | Reference |  |  |  | |  |
| II | 2.045 (0.002-21.416) | 0.609 |  |  | |  |
| III | NA | NA |  |  | |  |
| T stage |  |  |  |  | |  |
| T1 | Reference |  |  |  | |  |
| T2 | 0.248 (0.056-1.109) | 0.068 |  |  | |  |
| T3 | 0.366 (0.117-1.151) | 0.086 |  |  | |  |
| T4 | 0.841 (0.297-2.384) | 0.744 |  |  | |  |
| N stage |  |  |  |  | |  |
| N0 | Reference |  |  |  | |  |
| N1 | 0.328 (0.038-2.811) | 0.309 |  |  | |  |
| N2 | 0.901 (0.122-6.671) | 0.919 |  |  | |  |
| N3 | 1.438 (0.193-10.722) | 0.723 |  |  | |  |
| Stage |  |  |  |  | |  |
| II | Reference |  |  |  | |  |
| III | 0.855 (0.107-6.842) | 0.883 |  |  | |  |
| IVA | 2.413 (0.331-17.562) | 0.385 |  |  | |  |
| IVB | NA | NA |  |  | |  |
| IC cycle |  |  |  |  | |  |
| ≤2 | Reference |  |  |  | |  |
| >2 | 1.364 (0.540-3.447) | 0.512 |  |  | |  |
| IC regimen |  |  |  |  | |  |
| TP | Reference |  | Reference |  | |  |
| PF | 2.075 (1.084-3.971) | **0.028** | 2.393 (1.201-4.771) | **0.013** | |  |
| GP | NA | NA | NA | NA | |  |
| TPF | 1.967 (0.763-5.071) | 0.162 | 1.842 (0.707-4.797) | 0.211 | |  |
| Adjuvant chemotherapy |  |  |  |  | |  |
| No | Reference |  |  |  | |  |
| Yes | 1.065 (0.586-1.935) | 0.837 |  |  | |  |
| Induction platinum dosage |  |  |  |  | |  |
| <153.4 | Reference |  | Reference |  | |  |
| ≥153.4 | 2.107 (1.160-3.829) | **0.014** | 2.215 (1.177-4.166) | **0.014** | |  |
| Concurrent platinum dosage |  |  |  |  | |  |
| <201 | Reference |  |  |  | |  |
| ≥201 | 1.931 (0.964-3.868) | 0.063 |  |  | |  |
| Primary tumor volume after IC (mm^3^) |  |  |  |  | |  |
| <63.3 | Reference |  | Reference |  | |  |
| ≥63.3 | 1.782 (1.003-3.167) | **0.049** | 1.641 (0.899-2.997) | 0.107 | |  |
| Cervical lymph node volume after IC (mm^3^) |  |  |  |  | |  |
| <34.5 | Reference |  | Reference |  | |  |
| ≥34.5 | 2.604 (1.477-4.590) | **0.001** | 2.503 (1.399-4.476) | **0.002** | |  |
| NLR |  |  |  |  | |  |
| <2.7 | Reference |  |  |  | |  |
| ≥2.7 | 1.194 (0.680-2.097) | 0.537 |  |  | |  |
| PLR |  |  |  |  | |  |
| <209 | Reference |  | Reference |  | |  |
| ≥209 | 1.857 (1.060-3.255) | **0.031** | 1.263 (0.645-2.471) | 0.495 | |  |
| LMR |  |  |  |  | |  |
| <2.1 | Reference |  | Reference |  | |  |
| ≥2.1 | 0.550 (0.308-0.983) | **0.044** | 0.714 (0.363-1.406) | 0.330 | |  |
| EBV-DNA |  |  |  |  | |  |
| Negative | Reference |  |  |  | |  |
| Positive | 0.814 (0.442-1.499) | 0.509 |  |  | |  |
| PR rate |  |  |  |  | |  |
| ≤49% | Reference |  | Reference |  | |  |
| >49% | 0.472 (0.264-0.844) | **0.011** | 0.575 (0.318-1.041) | 0.068 | |  |
| IC: Induction chemotherapy; TP: Taxol + Cisplatin; PF: Cisplatin+5-Fluorouracil; GP: Gemcitabine + Cisplatin; TPF: Taxol + Cisplatin + 5-Fluorouracil; NLR: Neutrophil to lymphocyte ratio; PLR: Platelet to lymphocyte ratio; LMR: Lymphocyte to monocyte ratio; PR: Partial remission ratio; NA: Not available | | | | | |  |
